# Editorial: Reviews in heart valve stenosis and regurgitation

**DOI:** 10.3389/fcvm.2024.1425021

**Published:** 2024-05-27

**Authors:** Julio Garcia, Maurizio Taramasso, Mohammad Sarraf

**Affiliations:** ^1^ Departments of Radiology and Cardiac Sciences, University of Calgary, Calgary, AB, Canada; ^2^ Heart Valve Clinic, University Hospital Zurich, Zurich, Switzerland; ^3^ Department of Cardiovascular Medicine, Mayo Clinic, Rochester, MN, United States

**Keywords:** heart valve, valve stenosis, valve regurgitation, valve replacement, transcatheter valve

**Editorial on the Research Topic** Reviews in heart valve stenosis and regurgitation

## 1 Introduction

This Editorial introduces “Reviews in Heart Valve Stenosis and Regurgitation”. The aim of this research topic is to collect high-quality reviews and original articles to highlight recent advances in heart valve disease, while also emphasizing important directions and new opportunities for future investigation. We anticipate that these collected studies will stimulate discussion in the research community that will lead to best practice applications in clinical, public health, and policy settings. Nine manuscripts were taken into consideration for this research topic, all of which underwent a rigorous peer review process. The contributions are summarized in Table 1.

**Table 1:** Summary of the published studies.

Author	Main findings
Valve replacement	
Wang et al.	There are two main research hot topics in the literature: (1) internet-based anticoagulation management models and (2) novel strategies for managing frail patients.
Huang et al.	Destruction of the aortic annulus increases mortality and healthcare cost management.
Transcatheter valves	
Yu et al.	The persistence of cardiac conduction abnormalities in low-risk and younger cohorts remains a challenge.
Koren et al.	Valve degeneration is the main concern for younger patients undergoing TAVR.
Hayek et al.	Coronary obstruction remains the main challenge for low-risk patients.
Rumi et al.	Health technology assessment agencies have reached the same recommendations on the use of TAVI despite differences in clinical and economic settings.
Bocchino et al.	A multidisciplinary evaluation team is recommended for treating young low-risk patients.
Aortic regurgitation	
Tang et al.	Patients with severe complications of Behçet's disease and end-stage heart failure may benefit from TAVR.
Risk factors	
Liu et al.	Cardiovascular and cerebrovascular diseases share common risk factors.

## 2 Valve replacement

For patients with valvular heart disease (VHD), therapeutic intervention includes transcatheter and surgical approaches. Valve replacement is indicated as the primary treatment for VHD. However, the decision may be influenced by multiple factors including but not limited to the high risk of heart failure, rehospitalization, the risk of reoperation, and various complications that may affect the patient (1). Furthermore, postoperative management approaches often lead to heterogeneous outcomes (2). For this research topic collection, Wang et al. performed a bibliometric analysis of the management of patients undergoing valve replacement and its effectiveness in determining prognosis. Bibliometric analysis is mainly used as a tool to understand the state of the art of a specific topic. It also helps to identify “hot” areas of research. In this study, Wang et al. aimed to demonstrate a strategy to identify the best management plan, based on high-impact published research. The authors identified two main hotspots: (1) the integration of internet-based anticoagulation management models and (2) the need for novel strategies to manage patients with frailty. Huang et al. focused on the surgical management of infective endocarditis. This study reported that the destruction of the aortic annulus increases mortality and healthcare costs. The latter emphasizes the importance of pre-, peri-, and postoperative management to reduce mortality and morbidity rates in patients with aortic valve endocarditis. Early and accurate diagnosis remained the main challenge to be addressed in this cohort. Both studies highlighted the importance of management optimization and the growing interest in transcatheter aortic valve replacement (TAVR).

## 3 Transcatheter valves

Transcatheter interventions have become a valuable alternative for the treatment of VHD. They have been rapidly adopted by patients with severe aortic stenosis (AS). The continuous improvement in transcatheter valve design, surgical safety, and reduction in complication rates and perioperative mortality have increased its treatment adoption (3). However, challenges exist in the incidence of cardiac conduction abnormalities after TAVR procedures. Yu et al. reviewed the latest mechanisms, predictors, and types of conduction abnormalities after transcatheter aortic valve replacement (TAVR). The authors emphasize the challenge derived from the persistence of cardiac conduction abnormalities for wider application in low-risk and younger cohorts. A comprehensive preoperative, perioperative, and postprocedural assessment, including anatomical, electrophysiologic, and surgical risk factors is suggested. A critical aspect of TAVR implantation is valve mal-deployment, which may lead to an increased risk of thrombus formation (4). Koren et al. provided an overview of the mechanisms, prevention strategies, and treatment of leaflet thrombosis in TAVR. Several factors may cause this condition, with recent studies suggesting that blood flow shear forces may be implicated in promoting the thrombosis cascade (5). Preventive measures are suggested for high-risk patients by intensifying antithrombotic therapy. The use of TAVR in low-risk patients has increased with positive outcomes in comparison to surgical approaches (6, 7). The main concern for younger patients undergoing TAVR is valve degeneration over time. The valve-in-valve concept has been proposed as a solution. Hayek et al. reviewed this approach, which involves comprehensive management and surveillance of coronary obstruction, and stroke prevention. The primary goal is to define lifelong management in terms of the likelihood of further interventions. As the use of TAVR in elderly patients is well adapted and established, its use has been extended to low-risk patients such as those with symptomatic severe aortic stenosis. Rumi et al. aimed to analyze the different methods and interpretations of outcomes and their limitations. The authors reported a certain degree of heterogeneity in current recommendations, assessment, development, and evaluation. The financial evaluation favors the cost-effectiveness of TAVR compared with surgical aortic valve replacement. Bocchino et al. aimed to review the adaptation of TAVR to younger patients. The authors highlighted the need to change the decision-making process in the treatment of VHD and the need for multidisciplinary evaluation supported by a specialized team treating young low-risk patients.

## 4 Aortic regurgitation

Another important consideration for patients with aortic stenosis is the development of aortic regurgitation. Behçet's disease is associated with a high rate of postoperative complications and mortality. Patients with severe regurgitation often present with perivalvular leakage, pseudoaneurysm, and a poor long-term prognosis. Tang et al. aimed to summarize the evolution of surgical techniques for Behçet's disease and its symptoms leading to aortic regurgitation (8, 9). The authors pointed out that patients with severe complications and end-stage heart failure may benefit from TAVR. However, it is recognized that more evidence is needed to define adequate surgical management.

## 5 Risk factors

Finally, Liu et al. reviewed the risk factors related to intracranial and extracranial arteries and coronary stenosis. These contributions support the contextualization of the close interaction between cardiovascular and cerebrovascular diseases considering the most common risk factors.
